# Contraceptive Implant–Related Acute Ulnar Neuropathy: Prompt Diagnosis, Early Referral, and Management Are Key

**Published:** 2018-10-04

**Authors:** Ayman Saeed, Nitisha Narayan, Ankur Pandya

**Affiliations:** Plastic and Reconstructive Surgery Department, Queen Alexandra Hospital, Portsmouth, United Kingdom

**Keywords:** ulnar nerve, neuropathy, contraception, implant, complications

## Abstract

**Objective:** Subdermal contraceptive implants are a well-established method of contraception. Aside from common side effects such as irregular menstrual periods, headaches, and weight gain, rarer complications related to their insertion and removal have been reported. These include traumatic peripheral neuropathy after procedures to remove nonpalpable implants. Only 2 cases of ulnar neuropathy after insertion of a contraceptive implant have been described in the literature, one which resolved spontaneously and another in which postoperative recovery was not described. We report a case of acute ulnar nerve neuropathy in a patient postinsertion of a contraceptive implant who achieved symptom resolution after prompt referral and treatment at a specialist plastic surgery center. **Methods:** A 22-year-old, right-hand-dominant woman presented 1 day postinsertion of a contraceptive implant (Implanon) in her left arm with paresthesia along the ulnar distribution of her hand and forearm, as well as shooting pain on palpating the course of the ulnar nerve. Ultrasonography found the implant to be lying in the subfascial plane of the inner arm. **Results:** The implant was found lying in the perineurium of the ulnar nerve, causing ulnar nerve neuropathy. A review 3 months after removal of the implant showed near-complete resolution of her symptoms. **Conclusions:** Complications related to implantable contraceptives may lead to significant morbidity. Appropriate training for health care professionals administering the devices is essential, as well as early referral to a specialist center to prevent permanent damage.

Subdermal contraceptive implants are a well-established method of contraception. The 2 most common types are etonogestrel (Implanon, Nexplanon) and levonorgestrel (Norplant) implants.^1^ These consist of single and multiple rod-shaped implants, respectively, approximately 40 mm in length and 2 mm in diameter, that are inserted under the skin by health care professionals under local anesthetic. Typically, an incision over the medial aspect of the nondominant arm, 8- to 10-cm proximal to the medial epicondyle of the humerus, allows insertion.^2^^,^^3^ Newer models, such as Nexplanon, consist of a rigid tube preloaded in the needle of a disposable applicator for ease of release.^4^ Common adverse reactions associated with these devices include irregular menstrual periods, headaches, vaginitis, weight gain, and spotting.^1^ Rarer complications related to their insertion and removal have been reported, including traumatic peripheral neuropathy, after procedures to remove nonpalpable implants.^5^ Only 2 cases of ulnar neuropathy after insertion of a contraceptive implant have been described in the literature.^6^^,^^7^ Osman et al^7^ reported a transient self-limiting sensory ulnar neuropathy, whereas Ong et al^6^ described a patient with sensory and motor deficits 10 years after insertion of her implant. We report a case of acute ulnar nerve neuropathy in a patient postinsertion of a contraceptive implant who achieved symptom resolution after prompt referral and treatment at a specialist plastic surgery center.

## ULNAR NERVE ANATOMY

The ulnar nerve is located on the medial aspect of the upper limb, innervating muscles and skin of the forearm and hand. It originates from the medial cord of the brachial plexus and lies medially to the axillary and brachial arteries as it travels toward the antecubital fossa. In the middle of the arm, it pierces the intermuscular septum, runs across the medial head of the triceps, where it enters the groove between the medial epicondyle and the olecranon. It rests posteriorly on the medial epicondyle. It enters the forearm between the 2 heads of the flexor carpi ulnaris, before continuing along the ulnar side of the flexor digitorum profundus. Approximately 5-cm proximal to the wrist, it divides into a dorsal branch and a volar branch.^8^

## CASE REPORT

**Figure 1:**
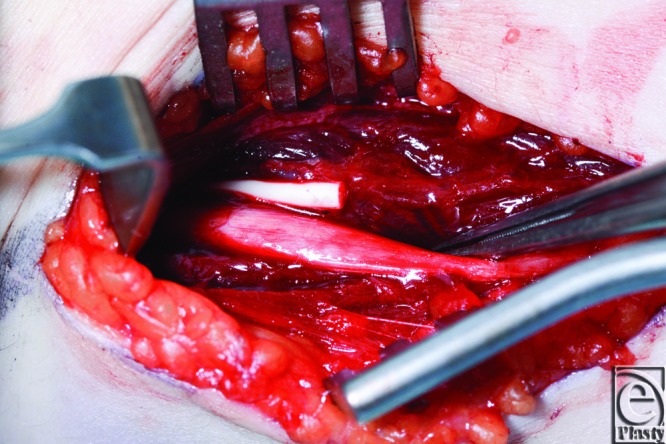
Contraceptive implant situated in the perineurium of the ulnar nerve.

A 22-year-old, right-hand-dominant woman was referred to our Plastic Surgery Department from her sexual health clinic 1 day postinsertion of a contraceptive implant (Implanon) in her left arm. It was explained that the implant was inserted by a nurse at the clinic who felt that the implant went in at “a slight angle, rather than superficial,” after the patient had flinched on insertion of the trochar. The subject experienced pain and paresthesia along her arm that had then subsided; however, she returned later that same day with worsening symptoms. On examination in the clinic, the implant was not palpable. The patient described paresthesia along the ulnar distribution of her hand and the forearm, as well as shooting pain on palpating the course of the ulnar nerve. Ultrasonography found the implant to be lying in the subfascial plane. On exploration in the operation theater, the implant was found lying in the perineurium, with the nerve itself intact (Fig 1). The medial intermuscular septum was released and the implant was removed in one piece without the need to repair any structures. She recovered well postoperatively. On review in the clinic 4 weeks later, she had persistent hypersensitivity of the dorsoulnar aspect of the distal forearm and reduced sensation in the ulnar digital and radial digital nerves of the little finger. The power of the intrinsic muscles in the hand was normal. Three months after removal of the implant, all her ulnar nerve functions apart from a slight residual sensory alteration had returned to normal.

## DISCUSSION

Traumatic peripheral neuropathy is a rare complication associated with contraceptive implants. Injuries to the median, ulnar, musculocutaneous, and medial cutaneous nerves of the arm have been reported.^5^^-^7^,^^9^^-^11 The majority of these cases have been associated with the removal of difficulty sited implants rather than insertion. There have only been 2 cases reported in the literature of ulnar neuropathy related to the insertion of a contraceptive implant,^6^^,^^7^ in contrast to 6 reported cases of ulnar neuropathy related to its removal.^5^^,^^9^^-^11 Ong et al^6^ described a patient experiencing intermittent left-hand numbness and weakness with associated claw-hand deformity over a 2-year period. Ultrasonography revealed a hyperechogenic structure impinging the ulnar nerve, which they attributed to a contraceptive implant inserted 10 years prior. The patient's recovery was not described in the report. In contrast, Osman et al^7^ described a young woman with ulnar nerve paresthesia postinsertion that resolved spontaneously. Our case highlights the importance of early referral of patients with iatrogenic neuropathies to a specialist center for early assessment and management. Prompt removal of the contraceptive implant in this case prevented the patient from developing permanent sequelae. It is also essential to inform patients of this potential risk and the symptoms they may experience in order to avoid long-term complications. Adherence to manufacturers’ instructions and the attendance of Royal College of General Practice (United Kingdom) accredited training programs should allow health care professionals to competently perform the insertion and removal of implants. It should also equip them with the knowledge of how to manage side effects when encountered.

## CONCLUSION

We conclude that insertion-related complications of implantable contraceptives, although rare, may lead to significant morbidity if not identified and dealt with promptly. Appropriate training for health care professionals administering the devices and well-informed patients should minimize the risk of nerve injury and early referral to a specialist center can prevent permanent damage.
